# Characterization of the influence of chlororespiration on the regulation of photosynthesis in the glaucophyte *Cyanophora paradoxa*

**DOI:** 10.1038/srep46100

**Published:** 2017-04-07

**Authors:** Masahiro Misumi, Kintake Sonoike

**Affiliations:** 1 Faculty of Education and Integrated Arts and Sciences, Waseda University, 2-2 Wakamatsu-cho, Shinjuku-ku, Tokyo, 162-8480, Japan

## Abstract

Glaucophytes are primary symbiotic algae with unique plastids called cyanelles, whose structure is most similar to ancestral cyanobacteria among plastids in photosynthetic organisms. Here we compare the regulation of photosynthesis in glaucophyte with that in cyanobacteria in the aim of elucidating the changes caused by the symbiosis in the interaction between photosynthetic electron transfer and other metabolic pathways. Chlorophyll fluorescence measurements of the glaucophyte *Cyanophora paradoxa* NIES-547 indicated that plastoquinone (PQ) pool in photosynthetic electron transfer was reduced in the dark by chlororespiration. The levels of nonphotochemical quenching of chlorophyll fluorescence was high in the dark but decreased under low light, and increased again under high light. This type of concave light dependence was quite similar to that observed in cyanobacteria. Moreover, the addition of ionophore hardly affected nonphotochemical quenching, suggesting state transition as a main component of the regulatory system in *C. paradoxa*. These results suggest that cyanelles of *C. paradoxa* retain many of the characteristics observed in their ancestral cyanobacteria. From the viewpoint of metabolic interactions, *C. paradoxa* is the primary symbiotic algae most similar to cyanobacteria than other lineages of photosynthetic organisms.

Approximately 2.5 billion years ago, cyanobacteria have evolved to use water molecule as electron donor for photosynthesis^1^. Change of photosynthetic machineries of cyanobacteria to those of anoxygenic photosynthetic bacteria is quite drastic: photosynthetic pigments are converted from bacteriochlorophylls to chlorophylls, two photosystems are connected in series to form linear electron flow, and water oxidizing complex is devised to split water molecules^2^. Ability of cyanobacteria to use water as electron donor for photosynthesis allows them to thrive on the entire surface of the earth. Evolution of photosynthesis after the emergence of the cyanobacteria looks less drastic, at least from a viewpoint of photosynthetic reaction centre. Structure of reaction centre complexes and the mechanisms of charge separation are almost identical between prokaryotic cyanobacteria and eukaryotic algae/land plants. This commonality of photosynthesis between two totally different domains of organisms can be explained by endosymbiosis theory^3^. All the plastids in eukaryotic cells originated from a single endosymbiosis event involving a eukaryote and a cyanobacterium^4^ in about one billion years ago^5^. After the event, three primary photosynthetic eukaryotes (green algae, red algae and glaucophytes) diverge from a common ancestor of eukaryotic photosynthetic organism.

Although photosynthetic reaction centre is well conserved among different algal groups as well as in cyanobacteria, their photosynthetic pigments and peripheral antenna systems are quite diverse^6^, which, in turn, result in the diversity of regulatory mechanisms for light harvesting systems^7^. Several different regulatory mechanisms are employed in cyanobacteria, which use phycobilisome (PBS) for their peripheral antenna. One mechanism is state transition, a distribution system of light energy from PBS to reaction centres, which is regulated by the redox state of plastoquinone (PQ) pool^8^. Another mechanism is energy dissipation system within PBS using orange carotenoid protein (OCP), which is activated by strong blue light^9^. Energy dissipation within PBS is also reported to be induced through the decoupling of PBS upon excessive irradiance or short heat stress^10^. Although approximately 80% of PBS-containing cyanobacteria use OCP^9^, eukaryotic algae, including red algae and glaucophytes that use PBS for peripheral antenna as cyanobacteria, lost OCP genes: instead, many eukaryotic algae acquire various energy dependent quenching mechanisms^7^. For example, the green alga *Chlamydomonas reinhardtii* uses light-harvesting complex stress-related protein 3 (LHCSR3) for energy dissipation system under high light condition^11^, while land plants use xanthophyll cycle^12^ and PsbS protein^13^ for the same purpose. Diatom, a secondary symbiotic alga in red lineage, uses diadinoxanthin cycle in place of xanthophyll cycle^14^.

The symbiosis producing eukaryotic algae must have triggered different kind of changes, i.e. interactions between chloroplasts derived from cyanobacteria and cytosol of host cells. Interactions between photosynthesis and other metabolisms, e.g. respiratory, nitrogen and carbon metabolism, are quite direct in photosynthetic prokaryotes such as cyanobacteria. Cyanobacteria do not have organelles and all the metabolic pathways can directly interact with one another within a cell. Particularly, photosynthetic electron transport and respiratory electron transport share several electron transfer components such as PQ, cytochrome *b*_6_/*f* complex and cytochrome *c*^15^,^16^. Respiratory NAD(P)H dehydrogenase (NDH)-1 complexes directly transfer electrons to PQ pool in photosynthetic electron transfer chain^17^,^18^. Thus, PQ pool is reduced in the dark by respiration in many cyanobacterial species^8^,^18^,^19^,^20^.

On the other hand, photosynthetic and respiratory electron transport chains are separated into organelles in eukaryotic cells; photosynthesis in chloroplasts and respiration in mitochondria. Nevertheless, the interaction between photosynthesis and respiration still exists in the form of chlororespiration^21^. In chloroplasts of land plants, plastidial NDH-1 complexes can transfer electrons from stromal NADPH to PQ pool^22^,^23^,^24^, while plastid terminal oxidases (PTOX) pull out electrons from PQ pool and transfer them to molecular oxygen^25^,^26^. Eukaryotic algae also possess PTOX^27^, but plastidial *ndh* genes have not been widely reported in plastid genome of eukaryotic algae^28^. In *Chlamydomonas*, however, a dehydrogenase component has been identified recently and was attributed to a type II NADPH dehydrogenase (NDA2)^29^.

The existence of chlororespiration in plastid may affect the redox state of PQ pool, just as in the case of the effect of respiration in cyanobacteria. In case of eukaryotic algae, however, the effect of chlororespiration on the redox state of PQ pool in the dark seems to be controversial. In case of land plants, the PQ pool is oxidized in the dark^30^,^31^. PQ reduction is known to induce the regulations of distribution system of light energy (state transition) but such regulatory change is not observed in the dark^32^. In eukaryotic algae, the redox state of PQ pool in the dark seems to be oxidized in some species^33^,^34^, but highly reduced in a few species^35^,^36^. For some cases, different research groups report different results for the same species^37^,^38^. As for glaucophytes, virtually no information is available.

Glaucophytes have plastids that are most structurally similar to the ancestral cyanobacteria among the three lineages of primary symbiotic algae. The plastids of glaucophytes, usually called as cyanelles, are thought as “living fossils“^39^ because the cyanelles keep several features of cyanobacteria such as PBS for peripheral antenna^40^, lack of membrane-intrinsic light-harvesting chlorophyll protein complexes (LHCs)^41^, peptidoglycan wall^42^ and carboxysomes, organelle-like polyhedral bodies involved in CO_2_ fixation^39^,^43^. These features, except for the presence of PBS, cannot be observed in the other two primary photosynthetic eukaryotes. On the other hand, likewise other algal plastid genome, genome size of cyanelle is small and approximately 1/10 compared to that of cyanobacteria^44^,^45^. In spite of these structural similarities between cyanobacteria and glaucophytes, recent comparative genomics and phylogenetic studies have not conclusively resolved the branching position of the glaucophytes, and the early branching history of the three primary photosynthetic lineages is still uncertain^46^,^47^. There is a report for photosynthetic activity of glaucophytes^48^ but there is no report on the regulatory aspects of photosynthesis: existence of chlororespiration in glaucophytes is not clear and its effect on the redox state of photosynthetic electron transport is totally unknown. For the understanding of the diversity of photosynthetic regulation and metabolic interaction among primary symbiotic algae, lack of information of these aspects in glaucophytes should be made up for.

Here we investigate the effect of chlororespiration on photosynthesis in the glaucophyte *Cyanophora paradoxa* NIES-547 through the measurements of chlorophyll fluorescence. The results clearly indicate that the effect of chlororespiration on photosynthesis in *C. paradoxa* is surprisingly similar to the interaction between respiration and photosynthesis in cyanobacteria. The chlorophyll fluorescence measurements also reveal that the main regulatory mechanism of the light harvesting systems is state transition even under photoautotrophic high light condition, just in the case of cyanobacteria. These results suggest that cyanelles of *C. paradoxa* retain many of the characteristics observed in their ancestral cyanobacteria. From the viewpoint of metabolic interactions, *C. paradoxa* is the primary symbiotic algae most similar to cyanobacteria.

## Results

### PQ pool is reduced in the dark in *Cyanophora paradoxa*

**Figure 1:**
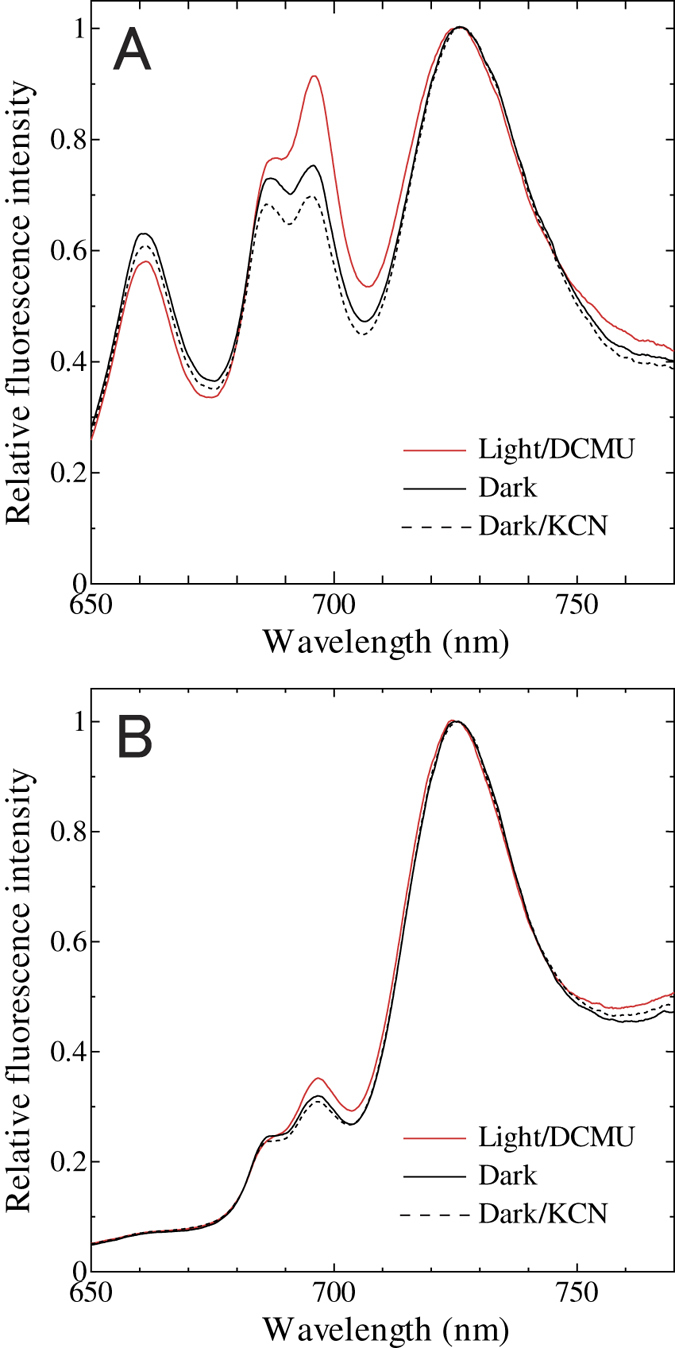
Chlorophyll fluorescence emission spectra determined at 77 K with phycocyanin excitation at 625 nm (**A**) or chlorophyll excitation at 435 nm (**B**). Black solid line, dark-adapted cells without any addition; black dotted line, dark-adapted cells in the presence of 1 mM KCN; red solid line, illuminated cells in the presence of 10 μM DCMU. Averages of spectra with three independent cultures are presented.

Effect of chlororespiration on photosynthesis should be reflected in the redox poise of PQ pool in the dark. Reduction of PQ pool induces state transition, resulting in more energy allocation to PSI, the extent of which could be estimated by chlorophyll fluorescence measurement of the cells at liquid nitrogen temperature. Upon PBS excitation at 625 nm of glaucophyte *C. paradoxa* cells, both PSI fluorescence (at 725 nm) and PSII fluorescence (at 685/695 nm) were observed reflecting the energy transfer from PBS to both photosystems (Fig. 1A). Illumination of the cells in the presence of DCMU fully oxidized the PQ pool and brought the cells to State 1, resulting in the high PSII fluorescence/PSI fluorescence ratio (F_695_/F_725_) of 0.904 ± 0.025 that reflects preferential energy allocation to PSII from PBS (Fig. 1A, red solid line). Dark acclimation of cells in the presence of KCN reduces the PQ pool through respiration in cyanobacteria^18^ or through chlororespiration in green algae^21^,^49^. This was also the case in glaucophyte: the cells of *C. paradoxa* were locked in State 2 with low F_695_/F_725_ ratio of 0.697 ± 0.013 (Fig. 1A, black dotted line). The simple dark acclimation of the cells in the absence of KCN resulted in the F_695_/F_725_ ratio of 0.750 ± 0.014 (Fig. 1A, black solid line). If we assume a liner relationship between the redox of PQ pool and F_695_/F_725_ ratio, approximately 70% of PQ pool would be reduced in the dark in *C. paradoxa. C. paradoxa* would have also the ability of spill-over type state transition, since much smaller but similar differences were also observed for the chlorophyll excitation at 435 nm (Fig. 1B).

In land plants, Fv/Fm, calculated as (Fm-Fo)/Fm, is widely used for the parameter representing the efficiency of PSII function, since this parameter could be simply determined from the room temperature fluorescence level of dark-acclimated samples (Fo) and that upon the saturating pulse (Fm). However, if state transition is induced in the dark acclimated cells of *C. paradoxa,* fluorescence level upon the application of saturating light pulse in the dark (Fm’_dark_) should be already quenched and lower than the level of true Fm determined under illumination in the presence of DCMU. This is the case and the level of (Fv’/Fm’)_dark_, i.e. “apparent Fv/Fm” level determined for the dark acclimated cells, was smaller than the true Fv/Fm level (Table 1). This quenching of the chlorophyll fluorescence in dark acclimated *C. paradoxa* cells was not relieved by the addition of nigericin, an ionophore that would collapse proton gradient (Table 1). Thus, the fluorescence quenching in the dark could not be ascribed to energy dependent quenching triggered by the proton gradient across the thylakoid membrane but to decrease of cross-section of PSII through state transition triggered by the reduction of the PQ pool. In other words, the PQ pool is reduced by chlororespiration, and PBS is functionally disconnected from PSII (i.e. being in State 2) in the dark acclimated cells of *C. paradoxa*.

**Figure 2:**
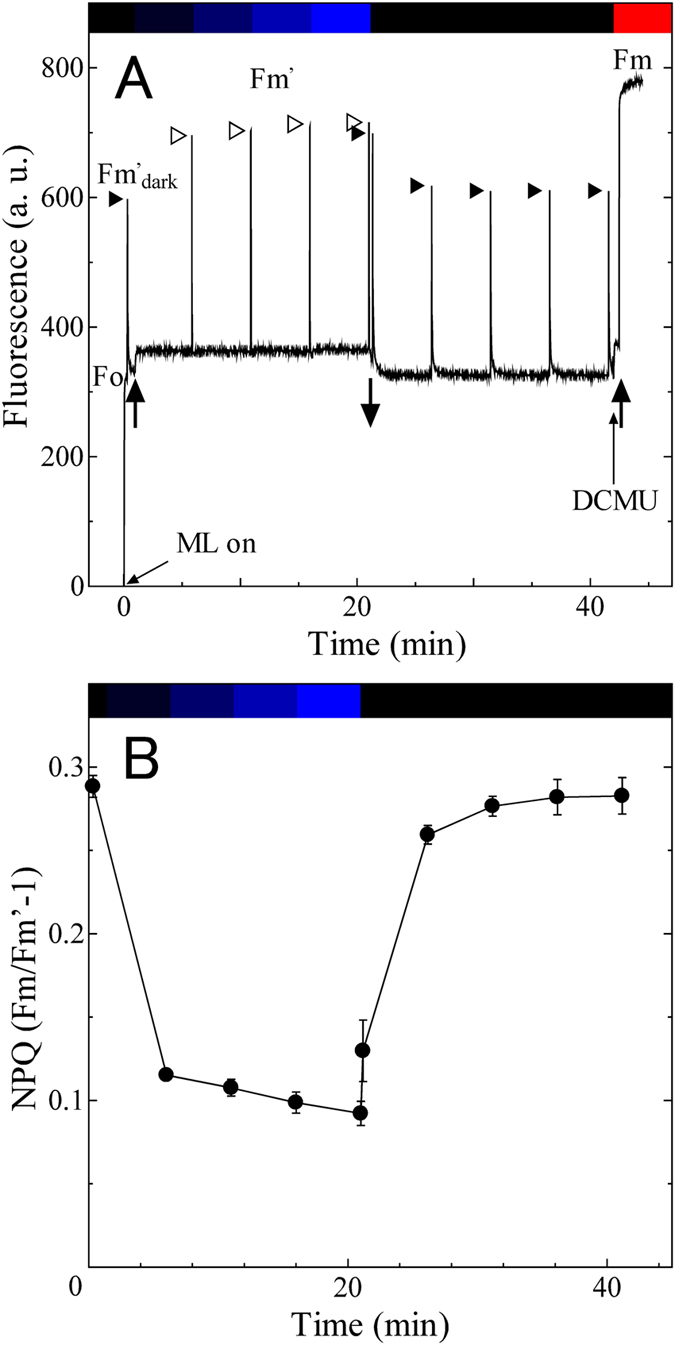
Quenching analysis of the chlorophyll fluorescence kinetics of *C. paradoxa* (**A**), and the change in NPQ calculated from the chlorophyll fluorescence kinetics (**B**). Actinic light was turned on at the time point indicated by solid upward arrows and off at the time point indicated by a downward arrow. DCMU was added at the time point indicated by a thin upward arrow. The bar on the top of the figure indicates illumination condition; dark (black), blue light (blue) or red light at 562 μmol m^−2^ s^−1^ (red). The change in the deepness of the blue colour represents different photon flux densities (44.6, 72.8, 145 and 288 μmol m^−2^ s^−1^) each applied for 5 min in step-wise manner. Averages of NPQ in three independent cultures are presented and vertical bars indicate standard deviation in panel B. See material and method for details.

PQ pool would be oxidized upon illumination of the *C. paradoxa* cells by blue light, since PSI is preferentially excited by chlorophyll-absorbing light, presumably due to very high PSI/PSII ratio in *C. paradoxa* (Fig. 1B). Under weak blue light at 44.6 μmol m^−2^ s^−1^, the level of Fm’ (Fig. 2A, the leftmost open arrowhead) was much higher than that in the dark acclimated cells (Fm’_dark_) (the leftmost filled arrowhead) and became closer to the Fm level determined in the presence of DCMU. Apparently, the chlorophyll quenching was reversed upon the oxidation of the PQ pool by weak blue light. On the other hand, the subsequent higher blue light illumination (72.8, 145 and 288 μmol m^−2^ s^−1^) did not cause any further changes in the levels of Fm’ (Fig. 2A, the remaining open arrowheads). The result suggests that energy dependent quenching is not induced even under strong blue light illumination in *C. paradoxa*. Under blue light, the levels of the nonphotochemical quenching (NPQ) parameter, calculated as Fm/Fm’−1^50^, decreased to one third of that in the dark (Fig. 2B). After turning off of the blue light, Fm’ was quenched to the initial Fm’_dark_ level again in subsequent five minutes (Fig. 2A, the remaining filled arrowheads and Fig. 2B), suggesting the reversible transition to State 2 in the dark, presumably due to the electron flow to PQ pool through chlororespiration.

### State transition is still the main photoregulatory mechanism even under high light for 4–5 minutes

**Figure 3:**
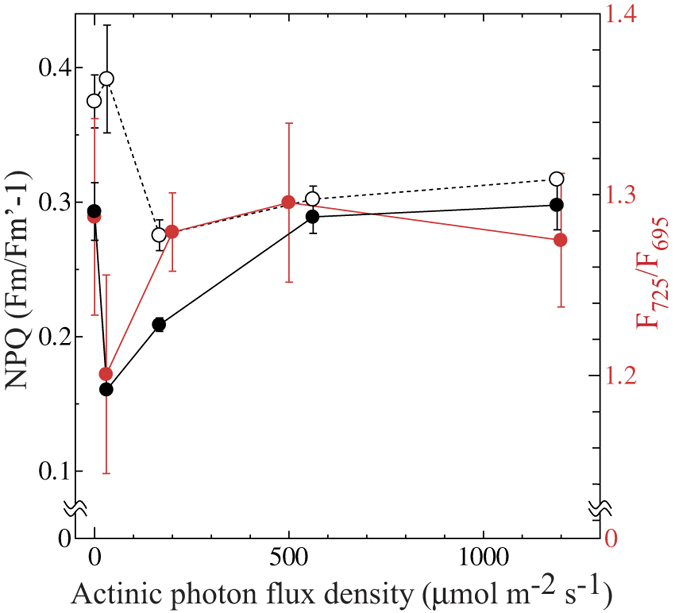
Red actinic light (peak at 660 nm) dependence of NPQ in *C. paradoxa* (black filled circles) and *Synechocystis* sp. PCC 6803 (open circles) at room temperature and white light dependence of state transition (red filled circles, corresponding to right vertical axis) estimated by the ratio of PSI fluorescence (725 nm) to PSII fluorescence (695 nm) determined at 77 K. Averages of at least three independent cultures are presented respectively and vertical bars indicate standard deviation.

In the case of cyanobacteria, not only blue light that preferentially excites PSI but also weak white or red light that excites both PSI and PSII are known to oxidize PQ pool. Since illumination by higher light reduces the PQ pool, NPQ values are high in the dark as well as under high light but low under growth light condition, resulting in the concave dependence on actinic light intensity^8^. This concave dependence of NPQ is observed in a wide range of cyanobacteria^20^, including a model cyanobacterium *Synechocystis* sp. PCC6803 (Fig. 3, open circles). We found that glaucophyte shows similar light dependence of NPQ (Fig. 3). When red actinic light was used, the levels of NPQ of glaucophyte cells were high in the dark and under high light, with minimum NPQ values around the actinic light at 31.5 μmol m^−2^ s^−1^ (Fig. 3, black filled circles). Under red actinic light, the main component of NPQ in cyanobacteria is shown to be state transition^8^,^20^. This seems to be also true for glaucophytes, since similar concave change was observed for the actinic light dependence of state transition estimated by relative chlorophyll fluorescence of PSI to that of PSII (F_725_/F_695_) determined at 77 K upon phycocyanin excitation (Fig. 3, red filled circles).

**Figure 4:**
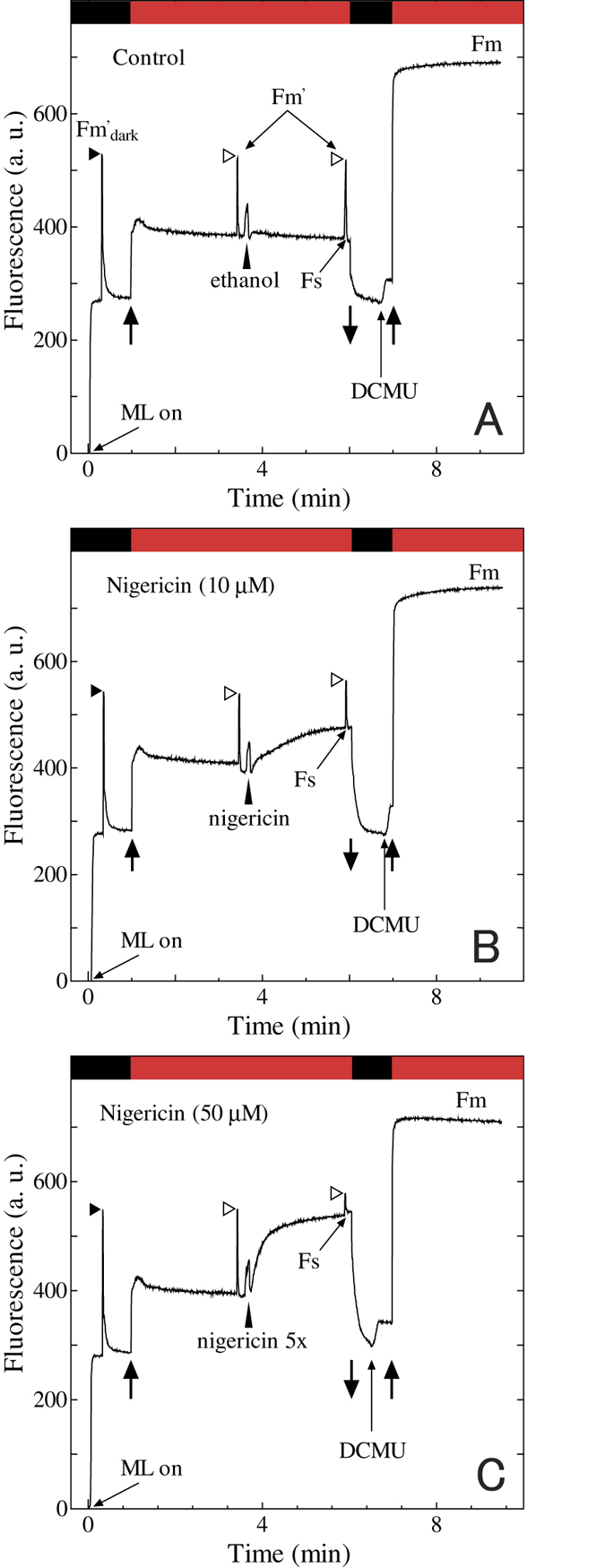
The effect of ionophore (nigericin) on chlorophyll fluorescence kinetics. After 2.5 min from turning on of red actinic light (solid upward arrow), saturating light was applied to obtain first Fm’. Soon after, ethanol for mock control (**A**) or nigericin (final concentration at 10 μM in the panel B experiment or at 50 μM in the panel C experiment) was added. After the level of fluorescence settled down to the steady state (Fs), saturating light was applied to obtain second Fm’. Then, red actinic light temporary turned off (solid downward arrow), and DCMU was added (thin upward arrow). Finally, red actinic light was turned on again and bring the cells to State 1 for the determination of Fm.

Apparently the main cause of the chlorophyll quenching was not energy dependent quenching but state transition. This assumption was further tested by the addition of an ionophore, nigericin, under high red light condition. After the addition of nigericin (10 or 50 μM) under strong red actinic light (562 μmol m^−2^ s^−1^), the level of Fm’ was only slightly affected even though the level of Fs gradually increased (Fig. 4B,C). The calculated NPQ in the presence of 10 μM nigericin (0.338 ± 0.030) was not significantly different from that before the addition of nigericin (0.406 ± 0.033) or that in the presence of mock control (ethanol) (0.361 ± 0.034). Thus, the low level of Fm’ under strong light for several minutes could be fully ascribed to state transition, not to energy-dependent quenching, similarly to the case of Fm’ in the dark presented in Table 1.

**Figure 5:**
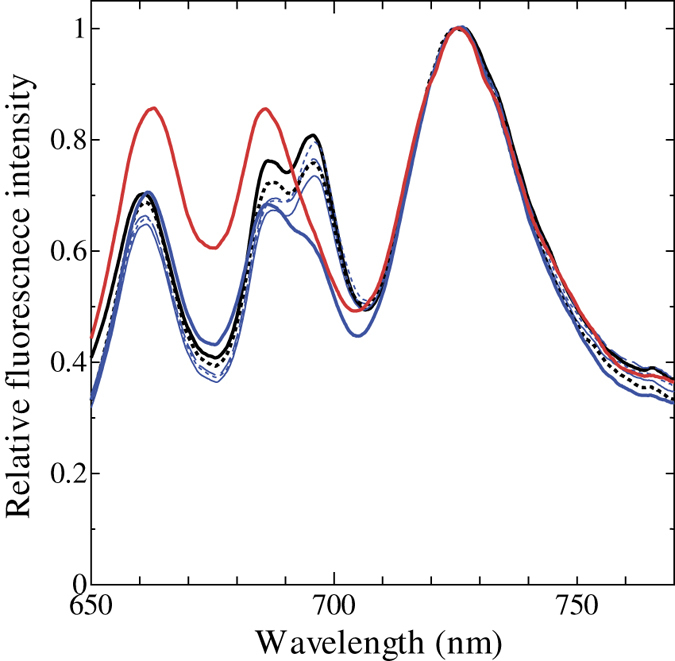
Effects of high light on 77 K chlorophyll fluorescence emission spectra with phycocyanin excitation at 625 nm. Black solid line, dark-adapted cells; black broken line, cells treated with illumination at 500 μmol m^−2^ s^−1^ for 180 min, blue lines, cells treated with 1200 μmol m^−2^ s^−1^ for 4 min (dotted line), 30 min (dashed line), 60 min (thin line) and 180 min (bold line); red line, cells treated with 2000 μmol m^−2^ s^−1^ for 180 min. Averages of spectra with three independent cultures are presented.

It must be noted, however, that the illumination of longer duration (180 min) with higher photon flux density (2000 μmol m^−2^ s^−1^) induced the reduction of chlorophyll fluorescence at 695 nm from PSII (F_695_) in compensation for the increase of 660 nm fluorescence from allophycocyanin and 685 nm fluorescence (Fig. 5, red line), suggesting the decoupling of PBS. Such increase was not observed in the cells treated with lower photon flux densities (1200 μmol m^−2^ s^−1^ and 500 μmol m^−2^ s^−1^) (Fig. 5, blue lines and black broken line, respectively). On the other hand, relative decease of F_695_ was observed upon illumination at 1200 μmol m^−2^ s^−1^ for 180 min but not at 500 μmol m^−2^ s^−1^, possibly reflecting some quenching mechanism working at 1200 μmol m^−2^ s^−1^ in PSII reaction centre.

### Red-shifted phycobilisome absorption in *C. paradoxa*

**Figure 6:**
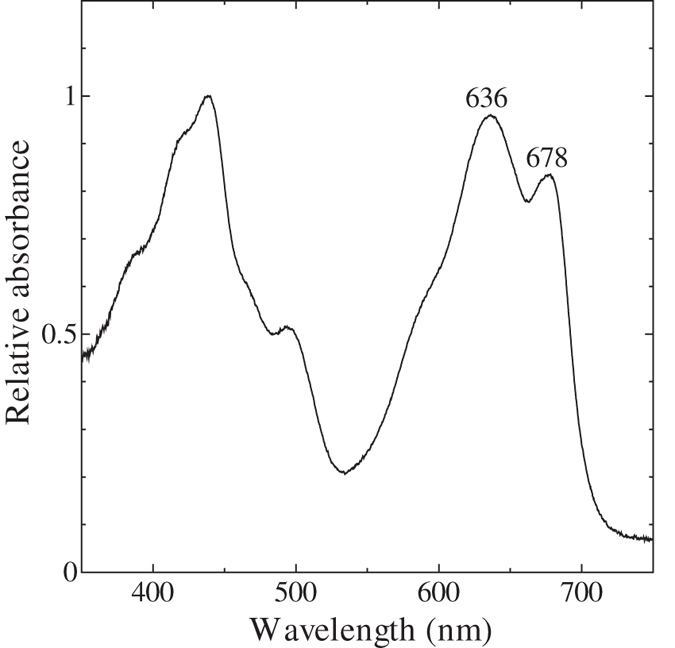
Absorption spectrum of the intact cells in the growth medium at room temperature. Average of spectrum with three independent cultures is presented.

Although the concave dependence of NPQ on the actinic light is similar between cyanobacteria and *C. paradoxa*, the level of actinic light that gives the minimum NPQ level seems to be different: While NPQ in cyanobacteria is minimum at the excitation around growth light level^8^,^20^ (see Fig. 3, open circles for *Synechocystis* sp. PCC 6803), NPQ in *C. paradoxa* gave minimum at 31.5 μmol m^−2^ s^−1^ that was approximately 1/6–1/7 of the growth light (Fig. 3). This difference could be partly ascribed to the difference in the absorbance of the photosynthetic pigments, since absorption spectrum of the intact cells of *C. paradoxa* at room temperature (Fig. 6) showed red shifted absorption peak of PBS at 636 nm, which is more close to the wavelength of the actinic light from red LED employed in this study (650 nm) compared with the absorption peak of cyanobacterial PBS (625 nm). The absorption spectra of *C. paradoxa* cells reported in the past also appears to indicate the presence of red shifted PBS^51^, although basic pigment composition (C-phycocyanin and allophycocyanin) of PBS is similar between cyanobacteria and *C. paradoxa*^52^.

## Discussion

PQ pool in prokaryotic cyanobacteria is known to be reduced in the dark due to the interaction between photosynthesis and respiration^8^, although there are some exceptions for certain cyanobacterial species that adapt to low light environments^20^. Here we show that the PQ pool is reduced in the dark in the eukaryotic glaucophyte, *Cyanophora paradoxa*. The level of NPQ in the dark was almost same as that under high actinic light condition, suggesting the highly reduced PQ pool in the dark (Fig. 3, black filled circles). The reduction of PQ pool in the dark was also supported by the chlorophyll fluorescence spectra determined at 77 K (Fig. 1A,B). Chlororespiratory electron flow to PQ pool must have substantial rate, since turning off of the blue actinic light that preferentially excites PSI triggered reduction of PQ pool in five minutes (Fig. 2A).

PQ pool of many eukaryotic photosynthetic organisms is known to be poised in a moderately oxidized state^30^,^33^,^34^ even though they do chlororespiration. On the other hand, PQ pool is reported to be highly reduced in the dark in a few eukaryotic algae such as chrysophyte *Ochromonas danica*^35^ and euglenophyte *Euglena gracilis*^36^. It is rather difficult to judge whether the highly reduced PQ pool is dependent on species because the two studies reporting high reduction of PQ pool employed photoheterotrophic growth condition. In *Chlamydomonas reinhardtii*, PQ pool is oxidized in the dark^34^ but becomes reduced upon addition of organic carbon source such as acetate^53^. Furthermore, the poise of PQ pool of *Chlamydomonas* in the dark can be affected by many other experimental conditions; inhibition of mitochondrial respiration^21^,^49^, nitrogen starvation^54^ and hyperosmotic condition^55^. Apparently, the redox poise of PQ pool in eukaryotic algae is dependent on environmental condition. In the case of *C. paradoxa*, however, PQ pool was already reduced under photoautotrophic condition without any stress (Fig. 1A,B) as in cyanobacteria. In terms of redox poise of PQ pool, *C. paradoxa* seems to be more similar to cyanobacteria than to other eukaryotic algae.

According to the model of chlororespiration^56^, the mechanism of PQ reduction is as follows: First, NAD(P)H is provided by metabolic reactions in stromal side of chloroplast. This NAD(P)H reduces PQ by some plastidial NDH complexes. In addition, like in cyanobacteria^57^, PQ might be also reduced by succinate through thylakoid succinate dehydrogenase (SDH). As for the source of NAD(P)H in glaucophyte, glycolytic pathway is a candidate. The cyanelle of glaucophyte have almost complete set of glycolytic enzymes except for hexokinase and phosphofructokinase according to a proteomic analysis^58^. Thus, at least, the second half of glycolytic pathway (3-phosphoglyceraldehyde to pyruvate) can produce NAD(P)H in cyanelle. In addition, the cyanelle have an isoenzyme of glucose 6-phosphate dehydrogenase (G6PDH) involved in the oxidative pentose-phosphate pathway (OPPP), and this isoenzyme is reversibly inhibited by dithiothreitol (DTT)^59^. Through this enzyme NAD(P)H can be produced by catabolic action in stromal side of cyanelle in the dark.

The import of organic substances to chloroplasts from mitochondria through cytosol is reported to be important for chlororespiration^60^,^61^. On the other hand, isolated cyanelles do not exhibit malate/oxaloacetate exchange activity^62^ so that cyanelles may not have malate valve to discharge stromal NADPH to cytosol/mitochondria as reducing equivalents. As a result, the stromal side of cyanelles could be more reduced than the plastids of green and red algae, and electrons may be more easily transferred to PQ pool. As for electron donor to PQ pool, cyanelle (plastid) genome do not have plastidial NDH complexes gene^28^, similar to most of eukaryotic algae but different from cyanobacteria and land plants. When we looked at *C. paradoxa* complete genome^46^, genes with moderate similarities with cyanobacterial (*Synechocystis* sp. PCC6803) *ndh* genes, i.e. *ndhI* (sll0520), *ndhK* (slr1280, sll8031) and *ndhM* (sll1623), and those with cyanobacterial *sdh* gene (slr1233) could be found. However, the localization of the product of these genes was not known. We also looked for the homologs of *Chlamydomonas* NDA2 gene in *C. paradoxa* genome but found only sequences matched to the part of the NDA2 gene.

More reduced PQ pool in *C. paradoxa* than that in other algae could be also brought about by the lower activity of PTOX. PTOX activity must be important as a determinant of redox poise of PQ pool in *Chlamydomonas*, since the knockout mutant of *Chlamydomonas* defective in PTOX2, a major oxidase involved in chlororespiration in this organism, shows the phenotype of reduced PQ pool in the dark^34^. It is suggested that PTOX has complex evolutionary history with several independent duplication events^27^. *C. paradoxa* genome contains only a sequence that shows low similarity to PTOX of red algae, green algae and cyanobacteria. On the other hand, genome of another glaucophyte, *Glaucocystis nostochinearum*, contains a gene with much higher similarity with red and green algal PTOX sequence. It would be worth to compare the redox state of PQ pool of *Glaucocystis nostochinearum* with other algal species in the dark, in order to see whether the PTOX activity is the universally determinant of the redox poise of PQ pool in the dark among eukaryotic algae.

The reduced PQ pool in the dark in *C. paradoxa* (Figs 1, 2 and 3) could be interpreted as a protective mechanism from photoinhibition of PSI. As the mechanism of PSI photoinhibition, it is proposed that active oxygen species, which are produced through the reduction of oxygen molecule in the acceptor side of PSI, directly destroys iron-sulphur cluster of PSI^63^. In other words, the combination of inefficient electron transport in the downstream of PSI and the presence of excitation pressure to PSI from non-downregulating PSII is the cause of PSI photoinhibition, which would be fatal problem for obligate photoautotrophs such as *C. paradoxa*. In order to avoid the photoinhibition of PSI, photosynthetic organisms have several layers of protection mechanisms such as cyclic electron flow and down regulation of PSII^64^. For example, PGR5-dependent cyclic electron transport is essential for protection of PSI under fluctuating light in *Arabidopsis thaliana*^65^. Cyclic electron transport is especially important in the transition period from dark to light, since Calvin-Benson cycle is inactivated in the dark, leading to the inefficient electron transport in the downstream of PSI^66^. Furthermore, the double mutant strain of *Chlamydomonas, Crpgrl1npq4* deficient in both cyclic electron flow and energy dependent quenching mediated by LHCSR3 is reported to be more susceptible to PSI photoinhibition than the single cyclic electron flow deficient mutant *Crpgr1*^67^. The down regulation of PSII activity seems to be essential for the protection of PSI from photoinhibition. *C. paradoxa*, which does not develop energy dependent quenching (Fig. 4), may have reduced PQ pool in the dark in order to decrease the reducing pressure to PSI upon onset of light illumination by downregulating PSII as well as by inducing state transition in advance. Although physiological function of chlororespiration is still under discussion^27^,^60^, it may serve for the photoprotective mechanism under fluctuating light through the reduction of PQ pool in the dark in *C. paradoxa*, and this may be also true for the PQ reduction in cyanobacteria through respiration.

During the course of evolution from cyanobacteria to green algae and finally to land plants, relative importance of state transition in light acclimation seems to decrease while that of energy dependent quenching seems to increase. In the case of green algae, about 80% of light harvesting capacity is controlled by state transition^68^, while only 20–25% of light harvesting capacity relies on state transition in land plants^69^. Here we show that the main mechanism of the light acclimation in *C. paradoxa* is state transition as discussed above. It would be reasonable to assume that the contribution of regulated energy dependent quenching to NPQ in *C. paradoxa* is negligible for the following three reasons. First, the NPQ level in high light condition was comparable to the level in the dark (Fig. 3), suggesting that no additional quenching mechanism is induced under high light condition at least for several minutes. Secondly, increase in the photon flux density of blue light illumination did not cause any further changes in the levels of Fm’ (Fig. 2A). It must be also noted that *C. paradoxa* does not have OCP genes^7^. Thirdly, the Fm’ level under high red light condition was largely unaffected by the addition of the ionophore, nigericin. Instead, the significant increase of Fs level was observed when ionophore was added (Fig. 4B,C). The cause of the increase of Fs level would be ascribed to the reduction of electron transfer components that is induced by the suppressed carbon assimilation due to ATP shortage that is, in turn, induced by the collapse of ΔpH by the ionophore. It must be noted that the extent of the energy dependent quenching is regulated by several factors: the photon flux density of growth light in green algae^70^,^71^ and growth phase in chromophyte alga *O. danica*^35^ as well as in cryptophyte alga *Guillardia theta*^72^. Even though we grew the cells of *C. paradoxa* under relatively high light to the exponential phase for the experiments, we cannot deny the possibility that this alga shows energy dependent quenching for some specific growth environment.

Allophycocyanin fluorescence at 660 nm relative to PSI fluorescence determined at 77 K increased (Fig. 5) presumably due to decoupling of PBS. Although decoupling of PBS is observed in cyanobacteria^10^ as well as in red algae^73^, our results indicate that the condition for the decoupling of PBS in Glaucophyte is rather nonphysiological for this alga, i.e. 2000 μmol m^−2^ s^−1^ for 180 min. On the other hand, the decrease of chlorophyll fluorescence at 695 nm was observed under less severe condition, i.e. 1200 μmol m^−2^ s^−1^ for 180 min, which is still rather harsh condition for this alga. Although this quenching of fluorescence may be comparable to the reaction centre-based quenching observed in many red algal species^33^,^74^,^75^,^76^, kinetics of induction is totally different between red algae and *C. paradoxa*: reaction centre-based quenching in red algae is induced very fast (saturating multi-turnover light pulse is enough to induce this quenching) while high light for several minutes is not enough to induce fluorescence quenching at 695 nm in *C. paradoxa*. Considering that *C. paradoxa* cannot grow under continuous light at 1200 or 2000 μmol m^−2^ s^−1^, decoupling of PBS and/or reaction centre-based quenching in *C. paradoxa* would be a kind of damage brought about by extreme high light rather than regulatory energy dissipation system.

Although red algae share many physiological characteristics with glaucophytes and cyanobacteria, red algae are different from glaucophytes and cyanobacteria in the metabolism of ascorbic acid^77^, which is known to be important for xanthophyll cycle^78^. *C. paradoxa* seems to be the only photosynthetic eukaryote that lacks any isoform of ascorbate peroxidase, and cellular concentration of ascorbate in *C. paradoxa* is reported to be either very low or null^77^. Thus, ascorbate-dependent xanthophyll cycle would be absent in *C. paradoxa*, which is in accordance with the lack of energy dependent quenching demonstrated here. Interestingly, cyanobacteria also do not appear to use ascorbate for photoprotection^79^,^80^. The strategy of photoprotection in glaucophyte would be quite different from that of red algae and might be similar to cyanobacteria.

## Material and Method

### Strain and growth conditions

*Cyanophora paradoxa* strain NIES-547 was obtained from the Microbial Culture Collection of the National Institute for Environmental Studies (Tsukuba, Japan). Liquid cultures of the *C. paradoxa* cells were grown at 26 °C in C medium^81^. Cell cultures were bubbled with filtered air under continuous illumination at 200 μmol m^−2^ s^−1^ from white fluorescence tubes. The typical doubling time of *C. paradoxa* in logarithmic phase (OD_750_ = 0.04–1.4) was 22.8 h (±0.20). Cells were sampled in the logarithmic phase at OD_750_ = 0.4–0.8.

Culture conditions for *Synechocystis* sp. PCC 6803 were similar to those for *C. paradoxa*, except for temperature (30 °C) and medium (BG-11)^82^. The typical doubling time of *Synechocystis* sp. PCC 6803 in logarithmic phase (OD_750_ = 0.03–0.76) was 6.23 h (±0.013). Cells were sampled in the logarithmic phase at OD_750_ = 0.18–0.31.

### Chlorophyll fluorescence emission spectra

Chlorophyll fluorescence emission spectra were determined at 77 K with a fluorescence spectrometer (FP-8500, JASCO, Japan) with a low temperature attachment (PU-830, JASCO, Japan)^18^. Cell suspensions were adjusted to a concentration of 2 μg chlorophyll ml^−1^ in growth medium. Chlorophyll concentration was determined by extraction with 100% methanol^83^. Prior to the measurements, the cells were dark-adapted for 15 min with or without KCN (1 mM) (Fig. 1A,B), illuminated by while light (30, 200 and 500 μmol m^−2^ s^−1^) for 4 min (Fig. 3), or illuminated by white light at 500 μmol m^−2^ s^−1^ for 180 min, at 1200 μmol m^−2^ s^−1^ for 4, 30, 60 and 180 min or at 2000 μmol m^−2^ s^−1^ for 180 min (Fig. 5) from a light source (PICL-NRX, NIPPON P-I). The effect of 10 μM DCMU was also tested for the measurements of cells illuminated at 500 μmol m^−2^ s^−1^ (Fig. 1A,B). The samples were excited by 625 nm light for phycocyanin excitation and 435 nm for chlorophyll excitation with excitation slit width at 10 nm. The fluorescence spectra were recorded with fluorescence slit width at 2.5 nm and resolution of 0.2 nm. The spectra were corrected for the sensitivity of photomultiplier and spectrum of light source using a secondary standard light source (ESC-842, JASCO, Japan). Chlorophyll fluorescence emission spectra were normalized at their respective maxima at around 725 nm.

### Chlorophyll fluorescence measurements by pulse-amplitude modulation

Chlorophyll fluorescence was measured by pulse-amplitude modulation with a fluorometer (WATER-PAM, Waltz, Germany). Cell suspension of *C. paradoxa* was adjusted to a concentration of 1 μg chlorophyll ml^−1^ in growth medium. The cell suspension was continuously stirred during the experimental procedure including the time for dark-acclimation to avoid oxygen deficiency. Cells in 2 ml liquid culture were dark-acclimated for 15 min and minimum fluorescence level (Fo) was determined with measuring light (peak at 650 nm). A pulse of saturating light (0.8 s) was given to dark-acclimated cells to determine Fm’_dark_. Subsequently, one of the following 3 types of the experiments was conducted. (1) Cells were illuminated by 562 μmol m^−2^ s^−1^ red actinic light (peak at 660 nm) for 2.5 min in the presence of 10 μM DCMU and saturating light was given to monitor the level of maximum fluorescence (Fm) (Table 1, Fig. 3), which is necessary to calculate NPQ that represents redox poise of PQ pool. Nigericin (final concentration at 10 μM) was added just before the dark-acclimation if necessary (Table 1). (2) Cells were illuminated by blue light (peak at 460 nm) at four different photon flux densities (44.6, 72.8, 145 and 288 μmol m^−2^ s^−1^), each applied for 5 min in step-wise manner, to monitor fluorescence under each steady state condition to investigate the effect of blue actinic light. At the end of each blue light illumination, the saturating light was applied to monitor the level of Fm’ under respective light conditions. Following the turning off of the blue light, the pulses of the saturating light were applied to monitor the recovery kinetics of Fm’ at 10 sec, 5 min, 10 min, 15 min and 20 min in the dark. Finally, Fm was obtained by the addition of 10 μM DCMU under red actinic light (Fig. 2A). (3) Red actinic light at respective photon flux densities (31.5, 167, 562 or 1190 μmol m^−2^ s^−1^) was applied to the cells for 5 min to monitor fluorescence under steady state condition to investigate the relationship between the photon flux densities of red actinic light and the levels of NPQ. At the end of red actinic light illumination, the saturating light was applied to monitor maximum fluorescence of the light acclimated cells (Fm’). Then, red actinic light was turned off and cells were relieved in the dark for 5 min from the effect of actinic light and the saturating light was given again. Finally, Fm was obtained by the addition of 10 μM DCMU under the red actinic light (Fig. 3). Experimental conditions for *Synechocystis* sp. PCC 6803 were identical to those for *C. paradoxa* (Fig. 3). To test the effect of ionophore, the cells were illuminated by strong red light (562 μmol m^−2^s^−1^), and we first obtained the level of Fm’ by a pulse of saturating light 2.5 min after the onset of illumination. Then, 2 μl of ethanol as a mock control (Fig. 4A) or nigericin (2 μl or 10 μl; final concentration at 10 μM or 50 μM, respectively) (Fig. 4B,C) was added, and 2.5 min later, a pulse of saturating light was applied again to obtain second Fm’ level. Fluorescence parameters were calculated as the following: Fv/Fm’_dark_ = (Fm’_dark_ − Fo)/Fm’_dark_, NPQ = Fm/Fm’ − 1^50^.

### Absorbance spectrum

Absorbance spectrum was determined with a spectrophotometer (V-650, JASCO, Japan) equipped with integrating sphere (ISV-722, JASCO, Japan)^18^ at room temperature. Absorbance of cell suspensions was determined in a cuvette with light path length of 5 mm. Absorbance spectrum was normalized at its maximum.

## Additional Information

**How to cite this article:** Misumi, M. and Sonoike, K. Characterization of the influence of chlororespiration on the regulation of photosynthesis in the glaucophyte *Cyanophora paradoxa. Sci. Rep.* **7**, 46100; doi: 10.1038/srep46100 (2017).

**Publisher's note:** Springer Nature remains neutral with regard to jurisdictional claims in published maps and institutional affiliations.

## Figures and Tables

**Table 1:** Photosynthetic parameters of *C. paradoxa.*

	Fv’/Fm’_dark_	Fv/Fm
control	0.457 (±0.021)	0.580 (±0.010)
Nigericin (10 μM)	0.480 (±0.013)	0.595 (±0.008)

(Fv’/Fm’)_dark_ was calculated as (Fm’_dark_–Fo)/Fm’_dark_. Values represent the average ± standard deviation with three independent cultures.
